# Implementing a Patient-Reported Outcome Dashboard in Oncology Telemedicine Encounters: Clinician and Patient Adoption and Acceptability

**DOI:** 10.1200/OP.23.00493

**Published:** 2024-01-11

**Authors:** Nisha A. Mohindra, Ava Coughlin, Sheetal Kircher, Alesia O'Daniel, Cynthia Barnard, Kenzie A. Cameron, Lisa R. Hirschhorn, David Cella

**Affiliations:** ^1^ Division of Hematology and Oncology, Department of Medicine, Feinberg School of Medicine, Northwestern University, Chicago, IL; ^2^ Robert H. Lurie Comprehensive Cancer Center, Northwestern University, Chicago, IL; ^3^ Department of Medical Social Sciences, Feinberg School of Medicine, Northwestern University, Chicago, IL; ^4^ Northwestern Memorial Health Care, Chicago, IL; ^5^ Division of General Internal Medicine, Department of Medicine, Feinberg School of Medicine, Northwestern University, Chicago, IL

## Abstract

**PURPOSE:**

Telemedicine provides numerous benefits to patients, yet effective communication and symptom assessment remain a concern. The recent uptake of telemedicine provided an opportunity to use a newly developed dashboard with patient-reported outcome (PRO) information to enhance communication and shared decision making (SDM) during telemedicine appointments. The objective of this study was to identify barriers to using the dashboard during telemedicine, develop implementation strategies to address barriers, and pilot test use of this dashboard during telemedicine appointments in two practice settings to evaluate acceptability, adoption, fidelity, and effectiveness.

**METHODS:**

Patients and clinicians were interviewed to identify determinants to dashboard use in telemedicine. Implementation strategies were designed and refined through iterative feedback from stakeholders. A pilot study of dashboard use was conducted from March to September 2022. Acceptability, adoption, and fidelity were evaluated using mixed methods. SDM was evaluated using the collaboRATE measure.

**RESULTS:**

One hundred two patient encounters were evaluated. Most patients (62; 60%) had completed some PRO data at the time of their telemedicine encounter. Most (82; 80%) encounters had clinician confirmation that PRO data had been reviewed; however, collaborative review of the dashboard was documented in only 27%. Degree of SDM was high (mean collaboRATE score 3.40; SD, 0.11 [95% CI, 3.17 to 3.63] out of a maximum score of 4). Implementation strategies focused on patient engagement, education, and remote PRO completion. Clinician-facing strategies included education, practice facilitation, and small tests of change.

**CONCLUSION:**

This study demonstrated that implementation of a PRO-based dashboard into telemedicine appointments was feasible and had acceptable adoption and acceptability by patients and clinicians when several strategies were used to engage end users. Strategies targeting both patients and clinicians are needed to support routine and effective PRO integration in telemedicine.

## Teaser

As telemedicine is increasing in oncology, we need to understand ways to bring patient-reported outcomes into that setting. This study found that engaging end users and presenting PROs in an interpretable and meaningful way through use of dashboard can help align clinicians with patient preferences and health status when they are not in clinic together.

## INTRODUCTION

Telemedicine provides numerous benefits for patients, including increased access to specialists, reduced travel time, and cost-savings.^1^ Although telemedicine has been shown to be equivalent to usual care in many settings, clinicians have voiced concerns about effective communication, lacking symptom evaluation, and missing physical cues from patients.^2-4^

**CONTEXT**

**Key Objective**
As utilization of telemedicine is increasing in oncology, we need to understand ways to bring existing interventions, such as patient-reported outcomes (PROs), into that setting.
**Knowledge Generated**
Through multicomponent implementation strategies focused on both patients and clinicians, we pilot tested using PROs during telemedicine appointments through use of a novel dashboard within the electronic health record. The dashboard compiles relevant PROs and clinical data into a single tab in the EHR, with easy-to-understand visual scoring, to enhance care decisions during clinical visits.
**Relevance**
By presenting PROs in interpretable and meaningful ways during telemedicine appointments, the dashboard may better align clinicians and patients with patient preferences and health status in a remote setting.

Patient-reported outcomes (PROs) are a powerful way to assess how patients are feeling and functioning on the basis of their own self-report.^8,9^ Use of PROs in oncology care has demonstrated improvements in quality of life, symptom control, and overall survival.^10-13^ Furthermore, use of PROs can enhance patient-clinician communication and patient-centered care.^14,15^ Despite this evidence, adoption of PROs in real-world settings has been limited, and use in telemedicine remains understudied.^16-20^

To increase adoption of PROs at our center, we developed an oncology PRO dashboard codesigned by a diverse group of stakeholders that was intended to launch in March 2020.^21^ The dashboard compiles patient-generated data (PROs and patient goals) and relevant clinical data into the electronic health record.^22,23^ The dashboard aims to align clinicians and patients to patient preferences and health status to facilitate shared decision making (SDM). Although the dashboard was developed before use of telemedicine at our institution, we had not conceptualized feasibility or implementation of the dashboard for telemedicine encounters. The objective of this study was to identify clinician and patient perceived barriers and facilitators to using the dashboard during telemedicine appointments, develop implementation strategies to address these barriers, and pilot test use of the dashboard in two oncology practice settings to evaluate acceptability, adoption, and fidelity. We also measured patient-reported SDM during telemedicine visits where the dashboard was used.

## METHODS

### Design

The study was conducted in two phases—formative evaluation and pilot study. The formative evaluation identified determinants to using the dashboard during telemedicine encounters and these results were used to develop implementation strategies. The pilot study evaluated acceptability, adoption, and fidelity of using the dashboard during telemedicine, as well as patient-reported SDM.^24^ The Template for Intervention Description and Replication and the Standards for Reporting Implementation Studies^25^ guidelines were followed for the pilot study.^26,27^ The study was approved by the Northwestern University Institutional Review Board (STU00214404) and the pilot study was approved by the Northwestern Memorial Hospital Cancer Quality Committee.

### Context, Setting, and Intervention

The PRO dashboard was implemented in June 2020 for in-person clinic visits in Thoracic Oncology and Gastrointestinal Oncology clinics. These populations were selected, given the high symptom burden associated with these diseases and engagement of clinician champions interested in enhanced symptom monitoring with PROs. Before the COVID-19 pandemic, these practices did not use telemedicine.

**FIG 1.**
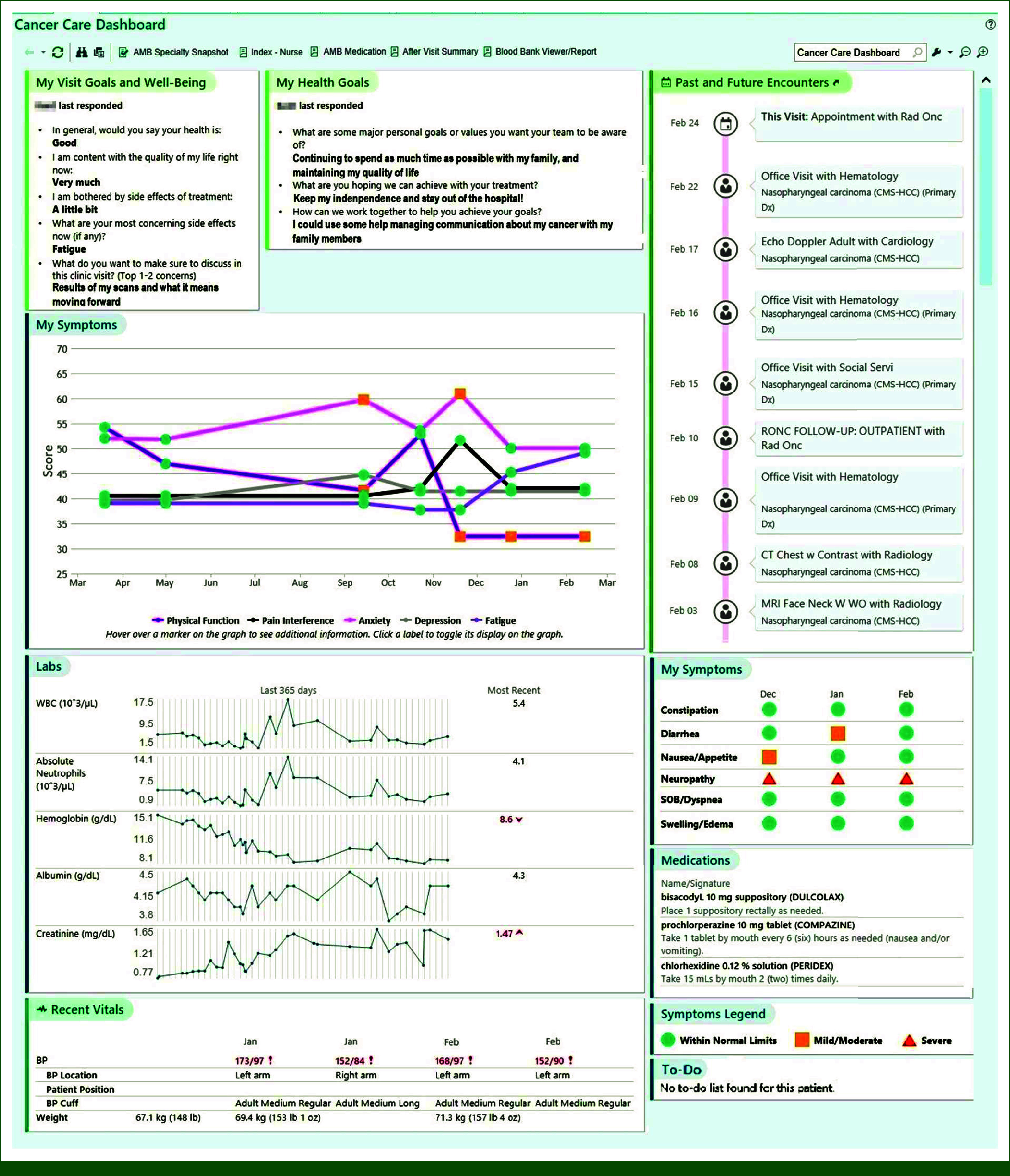
BP, blood pressure; CMS-HCC, Center for Medicare Services, hierarchical condition category; CT, computed tomography; SOB, shortness of breath.

Before this study, the oncology PRO dashboard was designed through a series of codesign sessions that included patients, care partners, clinicians from several disciplines, and health system leaders.^21^ The dashboard compiles patient-generated data (PROs and patient goals) with clinical data into a single tab in the electronic health record (EHR) (Fig 1). Patients respond to two types of questions: prompted questions (my visit goals and well-being and my health goals) and PROs of interest (Appendix Table A1, online only). Patients are guided with sample answers to each question to help generate ideas. Specific questions were developed with user testing during the dashboard coproduction process. PRO scores are derived from 2-item Patient-Reported Outcomes Measurement Information System assessments for each domain and scores are displayed in a color-coded severity scale and trended over time.^28,29^

**FIG A1.**
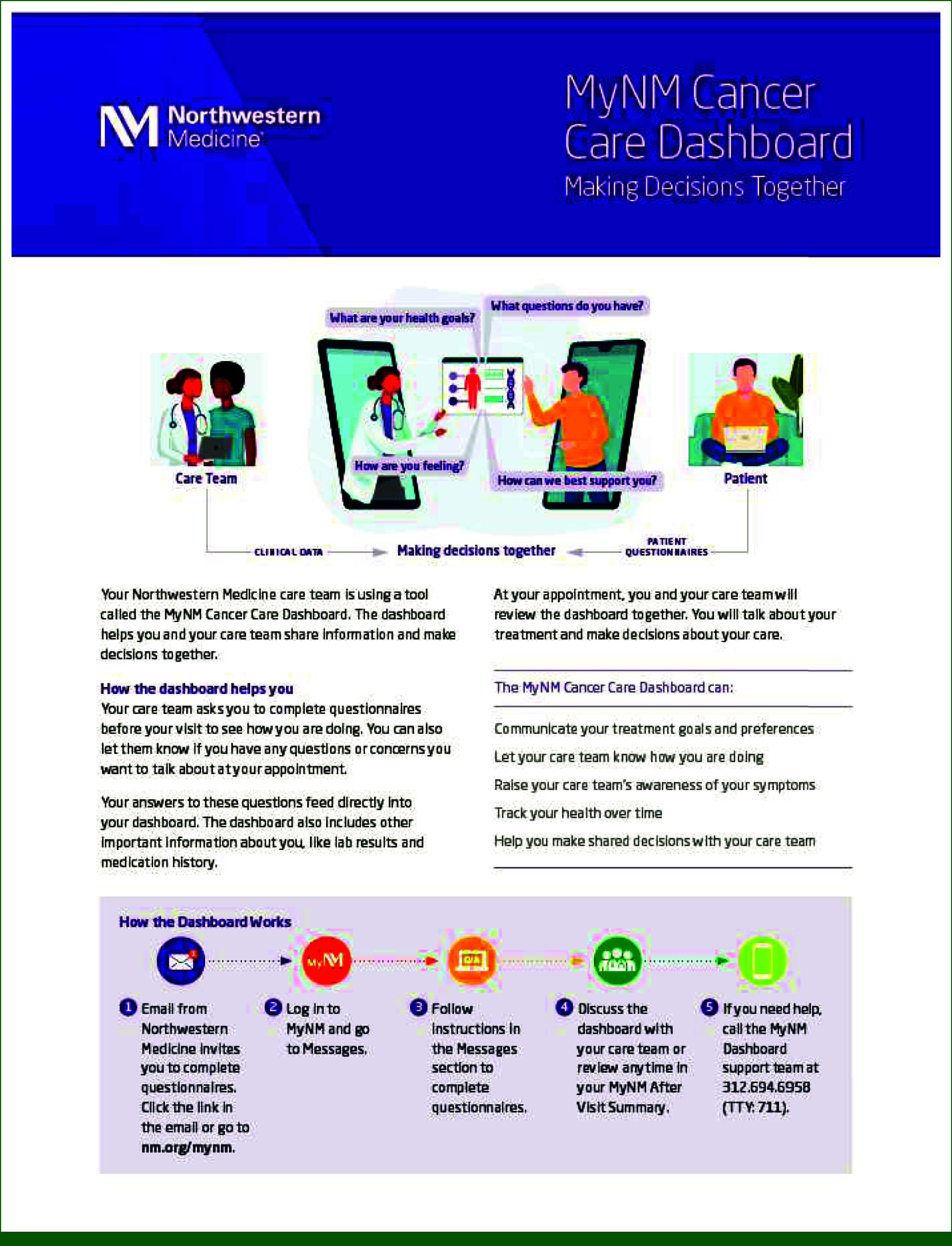
Infographic. NM, northwestern medicine.

Using the standard reporting framework from the EHR (EPIC Systems Corporation), we used a mix of available report components and custom-built components to create the dashboard in the local EHR system. Questionnaires were grouped into general well-being and disease-specific symptoms and displayed in different places for quick visualization. Clinicians access the dashboard through the EHR and data are automatically updated upon receipt of new data. Patients can review the dashboard PRO score reports in their After-Visit Summary on the online portal (Appendix Fig A1).

### Patient Identification

Eligible patients were identified through programming within the EHR (EPIC Systems Corporation) that screened for a diagnosis of stage IIIB/C/IV lung or GI cancers (esophageal, colorectal, gastric) in the problem list and/or a palliative-intent chemotherapy or immunotherapy treatment plan. Patients are sent a series of links to complete their PRO surveys, visit goals, and treatment goals 3 days before their appointment through the online MyChart portal (Trademark of EPIC Systems). Surveys are sent to patients every 30 days.

### Intervention

In this study, the PRO dashboard intervention was expanded to prompt patients to complete the assessments before telemedicine visits, and clinicians were trained to access the dashboard during telemedicine encounters.

### Part 1: Formative Evaluation

We conducted one-on-one interviews with clinicians (physicians, physician assistants, and nurse practitioners) and patients using semistructured interview guides to understand barriers and facilitators to using the dashboard during telemedicine. A convenience sample of clinicians was recruited (n = 11). Patients were recruited via referrals from clinicians, other clinical research studies, patient groups associated with the cancer center, and study flyers. Twenty-six patients were approached for recruitment, six of whom went on to complete an interview (Appendix Table A1).

Interviews were conducted with one of two trained interviewers: an oncologist (N.A.M.) with master's training in qualitative methods and a master's-trained project coordinator (A.C.) with experience in evaluating PROs in oncology. Separate interview guides were developed for patients and clinicians on the basis of the Technology Acceptance Model framework and the Consolidated Framework for Implementation Research (CFIR).^30,31^ Patient and clinician interview guides focused on understanding how users prepared for telemedicine visits and what challenges may arise if trying to complete PROs or use the dashboard during telemedicine visits.

Interviews were digitally recorded, transcribed verbatim, deidentified, and uploaded to Dedoose version 9. All participants provided consent to participate and were compensated for their time. Both an inductive approach and a deductive approach were used to describe themes related to barriers and facilitators of telemedicine appointments and to using the PRO dashboard during telemedicine appointments. Identified determinants were mapped back to CFIR. Coding was done iteratively, with constant comparison and review of codes until consensus was achieved to ensure rigor.^32^ Patient and clinician interviews were coded separately.

### Development of Implementation Strategies

On the basis of thematic analysis and the CFIR–Expert Recommendations for Implementing Change matching tool,^33,34^ implementation strategies were developed by the research team and iteratively refined based on stakeholder feedback. The research and clinical team used the quality improvement framework Define Measure Analyze Improve Control to update clinic workflows, define team roles for patient engagement and education, and prepare feedback reports related to implementation.

### Part 2: Pilot Study

The pilot study occurred from March 2022 to September 2022 and included patients with a scheduled telemedicine oncology appointment during that time frame. Patients were asked to complete a postvisit survey that included the collaboRATE questionnaire after their appointment.^35,36^ CollaboRATE is a three-item patient-reported experience measure of SDM.^24^ Mean scores for collaboRATE were calculated from surveys where responses were present for all three items. The survey also included questions related to ease of completing dashboard components, if additional assistance was needed or desired, and general feedback.

Patient adoption was measured through PRO completion rates (percent of eligible patients from sample with PROs present). Degree of PRO completion was also calculated—full completion (both goals and PRO surveys) versus partial completion. Clinician and patient acceptability were evaluated through qualitative feedback. Clinician acceptability was assessed through weekly-biweekly feedback sessions. Field notes of feedback from clinicians about their use of the dashboard during telemedicine appointments and patient surveys sent only after telemedicine appointments were reviewed by two members of the study team (A.C. and A.O.) and thematically analyzed. Fidelity of using the dashboard was evaluated through use of a smart phrase in clinic notes stating that the dashboard had been used in that clinical encounter (percent of notes during telemedicine with smart phrase) or through documentation of PRO use. Effectiveness was evaluated through collaboRATE scores for patients with eligible telemedicine visits.

Descriptive statistics were calculated for rates of PRO completion, degree of PRO completion, and clinical documentation of use of PRO data in our study population. Pearson chi-square tests and *t*-tests were used to compare patient characteristics of those completing or not completing PROs. Results were considered statistically significant at *P* values <.05. Statistical analyses were done using STATA version 17 (StataCorp LLC, College Station, TX). CollaboRATE scores were computed and interpreted according to developer guidelines.^24^

## RESULTS

### Clinician-Reported Facilitators and Barriers

Interviews were conducted with 11 oncology clinicians. Several facilitators were identified, and representative quotes are summarized in Table 1. Nearly all clinicians felt that the PRO score trends could serve as a quick reference of current and changing symptoms and could be helpful to assess symptom burden when patients are not in office and/or physical cues are absent. Furthermore, most felt that the disease-specific symptoms were clinically actionable, although some noted that this would not eliminate the need for a more complete review of systems. Most clinicians felt that the visit goals section could help obtain a more complete picture of patient concerns; some felt that this may reduce patient anxiety that not all questions would be answered during telemedicine visits.

**TABLE 1.** Representative Quotes on Barriers and Facilitator in Use of the Dashboard to Integrate PROs Into Telemedicine

Theme	Subtheme	Representative Quote	Participant ID
Clinician: perceived benefits	Visual aspect of dashboard helpful to communicate about changes in symptoms	“Well, I think one question we often ask patients is: “Compared to a few months ago, for example, is your energy any different?”. So, I could see with symptoms, whether it's nausea, diarrhea, fatigue, whatever. You can show the patient, just like what we can do with weight. Like if a patient said, “I'm losing this weight”, and then graphically you can show them you haven't lost any weight. I think that can give you a perspective if there is a change over time, so the patient can see how they responded before more graphically.”	C-07
	Dashboard as another mechanism to assess symptoms over telemedicine	“As far as telemedicine goes, I think this could be helpful for telemedicine, because what I miss with telemedicine is the actual face to face and you get a lot more out of the meeting, I think, in person than you do through telemedicine. So this could supplement some of that by actually outlining what their goals and wellbeing, as well as severity of symptoms.”	C-09
	Dashboard elements provide a more complete picture of patient needs	“I think it's just another way to really see what's been going on when they're not here in clinic, and how we can help them.”	C-05
Clinician: perceived barriers	Gaps in patient entry of PRO capture	“I think it's nice to be able to look at how symptoms have changed. It is nice to look at. And it would be certainly nice to show to patients, but again, it stems so much on the patient having the time to fill out those surveys that if they don't then it's not helpful.”	C-010
	Concern for added time during visits to educate about and discuss PRO data	“Oh, it'd be nice to have this information. What it's usually saying is it's falling on your shoulders to get it, and it's just too much for people. So, I'm all for having data readily available, but it's got to be something that's integrated into workflow. The other aspect of it, it may highlight particular concerns, so that, number one: you don't forget to address it, but it may actually change the whole focus of the encounter.”	C-07
	Feeling the need to address PROs in order for patients to continue to complete them	“So, for example, you'd want this to be front and center for the clinician who's going to engage with the patient. So you have to make sure that the docs are on the right page before you launch, because patients want to know that someone's looking at their data and someone's responding to it. Because why in the world would you continue to engage and report data, if particularly that happens multiple times, you report something and then nothing happens? Or no one addresses it, or no one even acknowledges that you've reported. Those are sort of the things that I would absolutely try to avoid.”	C-04
Patient: perceived benefits	Appreciate visualization of data and ability to trend health data	“I think that this is a super cool concept to have—I love the trendlines over time, and I love, you know, the ability—there's, like, all this data that we're always giving to the doctors, and it's awesome to, like, actually see it represented visually, and especially over time.”	P-010
	Deepened understanding of their symptoms	“I think something like this, where it's just like the basic symptoms, I think that's incredibly helpful, and then when I'm talking to my doctor, if it's something like this, and then they can kind of point out like, “Oh, hey, so this is what we're looking at, like, you know, you can see it's risen to a 70. That's of a concern to us,” or, “It's not a concern to us,” or, “We think, you know, it might be this but we're unsure. Do you think, do you have any insight as to why that's going on?””	P-012
Patient: perceived barriers	Difficulty identifying clinically relevant questionnaires	“I think we get bombarded, all their questionnaires and all their new results and we just get bombarded with so many other things that they don't really know that it's important.”	P-011
	Need for education and rationale for PROs	“You sort of like always need to explain the reason and benefit, especially for people who, you know, you can feel like a lab rat, uh, when it comes to, uh, cancer care and having to constantly be submitting, answering the same questions over and over again, blah-blah-blah-blah. Just explaining, like, “These are why we're asking the questions,” like, “This is what the cadence is going to be. This is what we hope to use it for,” like, “This is how it's going to be helpful to you and helpful to our relationship.””	P-012

Abbreviations: ID, Identification; PRO, patient-reported outcome.

In terms of barriers, clinicians noted concerns with capturing PROs when patients were not in clinic and that if PRO data were not present, clinicians would stop looking at the dashboard. Most mentioned that disease-specific PROs were not currently part of routine clinical care and that patients needed coaching and encouragement to complete them, but they did not have time during appointments to provide this support. Most clinicians felt heightened time constraints during telemedicine visits because of concerns with connectivity and that adding another task, such as reviewing the dashboard or providing education about PROs, may compete with existing visit-specific tasks. Some clinicians expressed concerns that if they did not address PRO data, it could affect patient willingness to complete PROs in the future.

### Patient-Reported Facilitators and Barriers

Of the seven patients who participated in the qualitative interviews, most patients felt that the dashboard would help them and/or their team better understand their health when they are not in clinic; however, a few felt that the data were overwhelming for self-review. All the patients found the visit goals and health goals sections valuable to facilitate communication team during telemedicine appointments.

In terms of barriers, patients reported needing education on what PROs were, how to complete the assessments, and that survey links were hard to find on the online portal. Most patients could not identify which surveys related to clinical care or feedback on clinical experience; however, most wanted to complete PROs if that information was valuable to their clinical team.

### Development of Implementation Strategies

**FIG A2.**
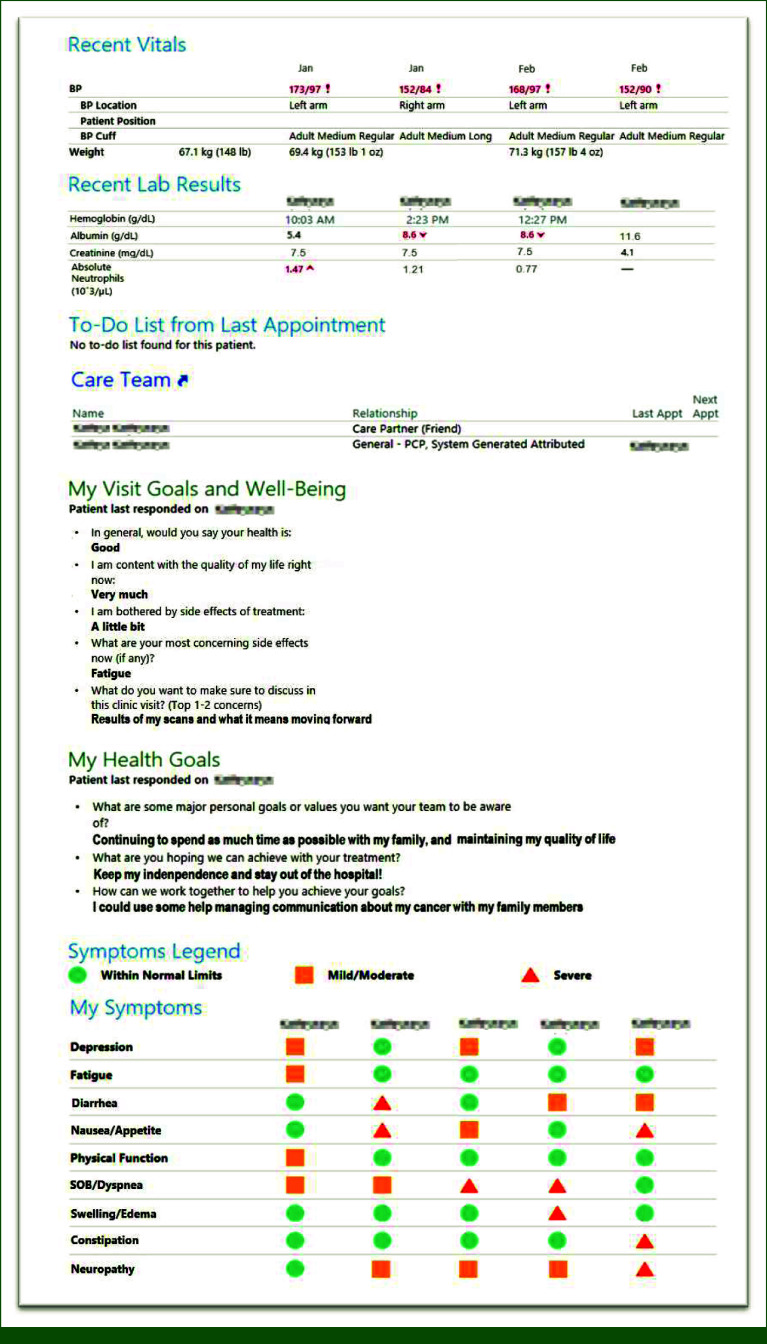
After Visit Summary. PCP, primary care physician; SOB, shortness of breath.

Several implementation strategies were developed and have been summarized in Table 2 per Proctor et al.^37^ Implementation strategies sought to (1) leverage the positive feedback loop between patients and clinicians who adopted the dashboard; (2) reduce clinician burden by creating workflows outside of clinic to capture and educate patients about PROs, (3) develop effective methods to capture PROs when patients were not in clinic, (4) educate and remind clinicians to use PRO data, (5) provide practice facilitation, and (6) assess small tests of change. Three patient-facing strategies were developed to improve capture of PROs, patient knowledge related to PROs/dashboard, and reduce clinician burden of having to do these tasks during their appointment. These included a personalized electronic message from the treating physician 2 days before the appointment; with an attached infographic (Appendix Fig A2); and creation of a part-time health outreach coordinator role to support patient outreach and PRO completion. Patients could access their PRO dashboard results through the After-Visit Summary (Appendix Tables A1 and A2 and Figs A1 and A2).

**TABLE 2.** Implementation Strategies to Support Use of PRO Dashboard in Telemedicine

Proctor Specification	Clinician-Focused Implementation Strategies						Patient-Focused Implementation Strategies		
	Educate Clinical Team	Prepare Champion	EMR Best Practice Alert	Cyclical Small Tests of Change (audit/feedback)	Practice Facilitation	Visual Reminder Card at Work Computer	Personalized Message From Clinician (prepare consumer to be active participant)	Infographic (prepare consumer to be active participant)	Outreach to Patients
Rationale/barriers	Lack of clinician experience with the dashboard and low perceived value of PROs	Lack of clinician experience with the dashboard	Reminder to use the dashboard	Reminder to use dashboard	Facilitate how to use dashboard	Reminder to use dashboard	Patients not completing surveys for PROs	Lack of understanding of PROs	Patients not completing surveys for PROs
Actor	Study team	Study team	EMR	Study coordinator	Study coordinator	Study team	Health outreach coordinator	Health outreach coordinator	Health outreach coordinator
Action	Educate	Dynamic training	Support clinician	Weekly report on patients PRO completion and documentation of use by clinician	Answer questions about dashboard ± information on dashboard	Support clinician adoption	Patient engagement	Patient education/engagement	Education/engagement/PRO completion by proxy
Target	MD/APP	MD/APP	MD/APP	MD/APP	MD/APP	MD/APP	Patients/care partner	Patients/care partners	Patients
Temporality	1 month before implementation and 1 week before implementation	Once, 2 weeks before implementation	Open at time of chart opening	1/wk × 12 weeks, then 2×/mo thereafter	1/wk × 12 weeks, then 2×/mo thereafter		2 days before each appointment	2 days before each appointment	Before each telemedicine visit (if PROs not completed on the day before appointment)
Dose	Two times	Once per clinical team member	Once/chart entry	16/study	16/study		Once/visit	Once/visit	As needed

Abbreviations: APP, advanced practice provider-nurse practitioners or physician assistants; EMR, electronic medical record; MD, medical doctor; PRO, patient-reported outcome.

### Pilot Study Results

The pilot sample included 102 eligible patients with a scheduled telemedicine outpatient oncology appointment during March 1, 2022-September 1, 2022. Over half of the patients were age 65 years or older (55%), 52% were female, 58% were non-Hispanic White, 66% were married, 55% had Medicare coverage, and 68% had a GI malignancy (Appendix Table A2). Descriptive data and implementation outcomes were derived from the EHR through the Electronic Data Warehouse.

A total of 62 (61%) patients had some PRO elements available at the time of their telemedicine encounter but only 25 (24%) had completed both goals for the visit and the PRO surveys. No statistically significant differences were noted between PRO completers versus noncompleters other than higher rates of completion in married patients (*P* = .03).

**FIG 2.**
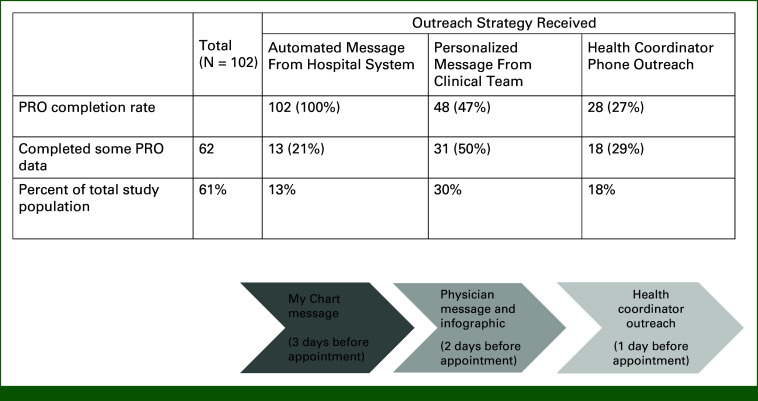
PRO, patient-reported outcome.

We evaluated at what time point PROs were completed relative to outreach strategy (Fig 2). Thirteen patients (13%) completed their PROs after the automated message from the NM system, while 49 patients (48%) completed PROs after receiving one or both additional outreach strategies. Four fifths (82 [80%]) of telemedicine encounters had a dot phrase noting that clinicians had reviewed PRO data but only 28 (27%) specifically used the smart phrase stating that the clinician had reviewed the PRO dashboard with their patient during the appointment.

Seventeen patients (17%) responded to the postvisit questionnaire. Thirteen patients (76%) found filling out the PRO assessments either easy or very easy and 10 patients (59%) reported not requiring additional help to complete their PROs. Twelve patients (71%) reported that completing their dashboard either completely or partially helped their conversation with their health care provider. Despite the education strategies, one patient expressed wanting to know why it was now necessary and another two patients noted that surveys did not address all their needs. Fifteen patients provided responses to all three collaboRATE measures, which is necessary for accurate scoring. Mean collaboRATE score during telemedicine visits was 3.40 (SD, 0.11 [95% CI, 3.17 to 3.63]) out of a maximum score of 4 (Table 3).^36^

**TABLE 3.** Evaluation of Shared Decision Making CollaboRATE Score

CollaboRATE Question	No. (respondents)	Mean Score (SD)
How much effort was made to help you understand your health issues?	17	2.9 (1.2)
How much effort was made to listen to the things that matter most to you about your health issues?	17	3.1 (1.2)
How much effort was made to include what matters most to you in choosing what to do next?	15	3.4 (0.5)
Composite score	15	3.4 (0.1)

NOTE. 0, no effort was made. 1, a little effort was made. 2, some effort was made. 3, a lot of effort was made. 4, every effort was made.

Abbreviation: SD, standard deviation.

Clinicians acknowledged the benefits of reviewing PRO data and patient goals before visits; however, they often missed this step when clinical volumes were high. Time constraints led physicians, more often than advance practice providers, to neglect dashboard data.

## DISCUSSION

This study demonstrates the feasibility of implementing a PRO dashboard into oncology telemedicine appointments. As in other studies implementing PROs into clinical care, a multicomponent plan for each stakeholder (patient and clinician) was used.^20^ However, as opposed to in-person care, our implementation strategies relied solely on remote methods for patient education and PRO capture. Despite improving visualization and interpretation of PRO scores, time constraints and competing clinical duties remained barriers for clinician uptake of the dashboard, echoing previous studies of PRO implementation in ambulatory oncology care.^38-40^ In our study, there was no difference in PRO completion by race and married patients were more likely to complete PROs and has been a factor associated with PRO completion in other studies.^41,42^

Clinicians noted omitting dashboard review when clinic volumes were high, suggesting that they may view these data as additive to clinic visits but not the highest priority during busy clinics. Currently, there are ongoing efforts to integrate PROs into value-based reimbursement plans, which may further drive prioritization of use and clinician uptake.^43^ Clinicians in our study noted feeling more time constraints during telemedicine visits than in-person visits and this may have reflected their relative inexperience with this modality of care at that time. Understanding the barriers by team member role is also important, as tailored training may be necessary to further increase adoption. For example, advanced practice providers focused largely on symptom scores and trends, as much of their visits address treatment tolerance. Physicians found the visit and health goals important and noted that these sections provided additional context for treatment decisions.

Because of the pilot design, patients could not access their dashboard until after their appointment in their After-Visit Summary. Expert groups recommend giving patients access to their PRO data to incentivize continued PRO reporting and increase the value of this information to patients.^44^ Providing these data to patients before their visit may be particularly helpful for telemedicine visits, where shared visualization of PRO data was not commonly used. When making that data available, health systems will need to ensure results are patient-friendly, interpretable, and can be used by patients to make care decisions. It was apparent from our study that patients needed more education on PROs and that these tools are screening tools to highlight which symptoms are bothersome, and to what degree, but are not meant to replace all interaction related to that symptom.

We found that high- and low-tech strategies helped achieve the goal of PRO availability at the time of the telemedicine visit. The number of patients completing PROs increased after receiving additional outreach through messaging and phone calls. Additive strategies allow for the most intensive strategies to be provided only to those who have not engaged with previous strategies, rather than delivering all strategies to all patients. Most patients were able to complete some PRO data and 25% completed all assessments. As noted in Figure 2, not all patients received prescribed outreach strategies. This was largely tied to the availability of the health outreach coordinator and the tight timeline of outreach, 1-2 days before appointment. The coordinator's role encompassed tracking PRO completion, sending the personalized messaging to applicable patients, and providing phone outreach. As this study only funded 20% of her time, competing demands or not reaching patients on allocated days reduced penetration of strategies. Future studies should consider automating personalized messaging to increase the number of patients reached.

Rates of PRO completion vary across the literature, ranging from 20% to 99%, and depend on types of patient engagement, specialty, number and type of PRO of assessments, and rationale for use.^45-48^ Clinical practices with PRO completion rates >50% have been considered successful, and our completion rate of 60% is noteworthy, given that in-person education and completion were not options.^47^ PRO completion rates in this study also compared favorably with historic PRO completion rates at our cancer center (approximately 20%) when used in standard-of-care practice.

PROs must be efficient and easy for patients to complete. The online patient portal had a somewhat burdensome and confusing interface, such that each section of the dashboard had a different link for completion. Patients reported it was not clear that each link contained separate questionnaires. Furthermore, we continue to work to understand clinically meaningful frequencies at which each assessment is needed to affect clinical care. Data around optimal frequency for questions such as goals of care are lacking. Frequency of assessments must weigh potential benefit relative to patient burden.

Our study had several limitations inherent in a pilot study. It was designed as an adaptation of an ongoing study of dashboard use during in-person visits and the results from that study are forthcoming. Although not a true comparator arm for the current study, understanding the differences between facilitators for in-person visits and telemedicine visits will be important to evaluate for larger-scale implementation in both settings. The intervention was done in two specialty oncology practices within an academic medical center; generalizability to other settings may be limited. Our formative evaluation and postvisit survey had limited patient responses, including the SDM measures. Most participants in the pilot were of White, non-Hispanic background, which may be due to PRO surveys in our study being only administered in English. We had to rely on use of smart phrases to note if PROs were reviewed by the clinician or by the clinician and patient, which may have underestimated or overestimated actual use and in what setting. The availability of two dotphrases may have been overly complex for the pilot study. Confirmation in larger and more diverse patient populations will be useful.

In conclusion, this study demonstrated that implementation of a PRO-based dashboard during telemedicine appointments was feasible and had acceptable adoption and acceptability when several strategies were used to engage end users. A multicomponent plan delivered reasonable PRO completion before telemedicine appointments and the presence of PRO data facilitated the use of the dashboard by clinicians. Despite their appeal and potential, effective integration of PRO dashboards into clinical workflow remains challenging and further study is warranted.

## APPENDIX

**TABLE A1.** Participant Demographics From Formative Evaluation

Clinician	Total (n = 11)
Physician, No.	7
Practice, years, No.	
1-5	2
5-10	5
>10	4
Female, No. (%)	8 (73)

Patient/Care Partner	Total (n = 9)
Patients, No. (%)	7 (78)
Age, years, No. (%)	
<45	3 (33)
45-70	4 (44)
>70	2 (22)
Female, No. (%)	7 (78)
Race	
White, No. (%)	4 (44)
Black/African American, No. (%)	1 (11)
Asian, No. (%)	3 (33)
Other, No.	0
Prefer not to answer, No. (%)	1 (11)
Report living <25 miles from cancer center, No. (%)	6 (67)

**TABLE A2.** Demographic and Clinical Characteristics of Pilot Study Population

	Total (N = 102), No. (%)	PRO Data (n = 62), No. (%)	No PRO Data (n = 40), No. (%)	*P*
Age category, years				.71
18-64	46 (45)	30 (48)	16 (40)	
>65	28 (27.5)	16 (26)	12 (30)	
>75	28 (27.5)	16 (26)	12 (30)	
Sex				.75
Female	53 (52)	33 (53)	20 (50)	
Male	49 (48)	29 (47)	20 (50)	
Race				.09
Non-Hispanic White	59 (58)	41 (66)	18 (45)	
Non-Hispanic Black	15 (15)	6 (10)	9 (23)	
Asian	7 (7)	5 (8)	2 (5)	
Other/declined	21 (21)	10 (6)	11 (28)	
Marital status				**.03**
Married	67 (66)	41 (66)	26 (65)	
Not married	31 (30)	21 (34)	10 (25)	
Unknown	4 (4)	0 (0)	4 (10)	
Insurance coverage				.37
Private	45 (44)	30 (48)	15 (38)	
Medicare	56 (55)	31 (50)	25 (63)	
Medicaid	1 (1)	1 (2)	0 (0)	
Malignancy type				.68
Lung cancer	33 (32)	21 (34)	12 (30)	
GI cancer	69 (68)	41 (66)	28 (70)	

NOTE. Values in bold indicate findings with *P* < .05.

Abbreviation: PRO, patient-reported outcome.
